# Discovery of Blood-Based Proteins That Mark Benzo[a]pyrene Modulation of Autoimmunity

**DOI:** 10.3390/ijms262010242

**Published:** 2025-10-21

**Authors:** Kameron Kennicott, Yilin Nie, Yun Liang

**Affiliations:** Departments of Physiology and Pharmacology and Toxicology, Michigan State University, East Lansing, MI 48824, USA

**Keywords:** autoimmune diseases, environmental exposure, benzo[a]pyrene, molecular mechanism

## Abstract

Environmental pollutants are thought to shape our immune landscape and drive the rise in autoimmune disease incidence worldwide. However, the molecular underpinnings of environmental impact on autoimmunity remain elusive and a quantitative measurement for immune dysfunction as a result of environmental exposure is yet to be developed. To this end, we have performed a discovery study to identify blood-based, immune-associated proteins regulated by benzo[a]pyrene (BaP) using the autoimmune-prone murine model MRL. We report the upregulation of autoimmune-associated cytokines including IL1a and IFNg by BaP, months before the manifestation of autoimmune phenotypes. Additionally, the increased levels of proteins such as IL16, IL22 and SNCA in male MRL mice upon BaP exposure may be a molecular link to the increased risk in end organ damage in subsets of autoimmune disease patients. Further comparison with the transcriptomic analysis of BaP-stimulated skin and lungs suggests distinct patterns of immune regulation in peripheral organs versus blood. Altogether, our study supports the need for the early detection of BaP-induced immune changes for the prevention and management of autoimmune diseases and provides leads for the future development of these blood-based biomarkers.

## 1. Introduction

Autoimmune diseases, commonly characterized by the loss of tolerance and the attack of self-organs by one’s immune systems, are becoming dramatically more prevalent in many parts of the world [1,2,3,4,5,6]. Environmental agents, which account for an estimated seventy percent of autoimmune disease risk, are thought to drive the rise in autoimmune disease incidence [1,2,3,4,5,6]. In comparison, genetic factors are thought to contribute to approximately thirty percent of disease risk [1,2,3,4,5,6]. Therefore, it is critical to understand how environmental pollution causes immune dysfunction and autoimmunity.

It is well-accepted that environmental factors including mercury (Hg), pesticides, pristane, smoking, silica and trichloroethene are linked to autoimmune diseases [1,2,3,4,5,6]. For example, pristane is known to cause lupus-like disease in susceptible mice characterized by Interferon (IFN)-I-dependent autoantibody production and inflammatory cytokines [7,8]. In murine models, inorganic Hg leads to the autoimmune phenotype, which is a novel model of systemic autoimmunity for its independence from type-I IFN [9,10,11]. Pesticide use is associated with higher levels of antinuclear autoantibodies (ANA) as well as an increased secretion of cytokines including TNFa, IFNg, IL2 and GM-CSF [12,13,14]. Epidemiological study supports the association between smoking and risk of systemic lupus erythematosus (SLE), which mechanistically may involve increased ANA, inhibition in Treg activity, and enhanced expression of inflammatory mediators [15,16,17]. Polycyclic aromatic hydrocarbons (PAH), a group of compounds produced from the incomplete burning of organic materials including fuel and tobacco, and which include benzo[a]pyrene (BaP), have been found to associate with risk of late-stage rheumatoid arthritis (RA), using urinary biomarkers of RA and adjusting for age, sex, race, educational level, marital status, smoking, BMI, physical activity, energy, diabetes, and survey cycle [18]. The disease severity of systemic lupus erythematosus (SLE), measured by the systemic lupus erythematosus disease activity index (SLEDAI), correlates positively with smoking status after adjusting for all covariates including ethnicity, educational level, income level, alcohol use, age of onset of SLE, current age, mean duration of SLE, marital status, and hydroxychloroquine therapy [19,20]. The levels of PM_2.5_, particles that often associate with PAH in air pollution, are found to increase risk of SLE using SLE diagnostic codes (ICD-9-CM code: 710.0) with additional review [21]. In animals, the PAH 1-aminoanthracene has been found to increase type-1-diabetes disease features including blood glucose and pancreatic inflammatory cytokine levels [22]. The levels of PAH in the environment vary greatly, with documented examples for BaP including 786 ng/m^3^ in cafeterias where frying occurred and 100 μg/m^3^ in aluminum production [23]. Additionally, humans intake significant amounts of BaP from smoking, food and water [23].

While there is increasing evidence linking environmental agents to various types of autoimmune diseases, significant knowledge gaps still remain regarding the exact mechanisms by which environmental factors contribute to disease etiology. Additionally, whether environmental exposure is one of the early events that trigger immune dysregulation is considered one of the most challenging yet critical aspects of autoimmune study [4].

To address these knowledge gaps, we studied the effect of exposure on the MRL mouse strain. The MRL mouse is commonly used as a control strain for MRL-*lpr*, which carries an additional *Fas*^lpr^ mutation and shows the accelerated development of systemic autoimmunity [24]. Despite carrying the normal *Fas* gene, MRL mice exhibit multiorgan, lupus-like disorders including glomerulonephritis, arthritis, skin rash, cerebritis, and anti-dsDNA antibodies at a late stage (~18 months; corresponding to ~56 years of age in humans) [25]. In our study, we used young MRL mice (at 2 months of age), aiming to understand whether environmental agents such as BaP can trigger immune dysfunction early, long before the manifestation of lupus-like phenotypes.

Our previous work has shown that BaP causes a drastic reduction in cell-type diversity in various organs including kidneys and lungs in MRL mice [26]. Furthermore, there is a male-biased induction of angiogenesis genes following exposure to BaP, which may help explain the increased likelihood of disease progression to lung cancer in male lupus patients [26,27]. However, the molecular changes in the MRL blood post-BaP exposure remains undefined.

Here we have performed multi-analyte proteomic analysis of blood from the BaP-exposed versus control MRL mice, using a novel proteomic method based on proximity extension technology [28]. We report the upregulation of specific cytokines including IL1a, IL16, IL22, IL33 and IFNg by BaP in these autoimmune-prone mice. Intriguingly, there is an increase in SNCA (alpha-synuclein) and p-SNCA in BaP-treated MRL mice, supporting a suspected link between autoimmune diseases and neurological disorders such as dementia [29]. In contrast, the analysis of skin and lungs from these mice suggests the downregulation of immunostimulatory genes. Our results indicate that the exact molecular effects caused by BaP are tissue-specific, and may help explain the paradoxical combination of immunostimulatory and immunosuppressive effects in autoimmune settings [18,26,30,31,32,33]. Our study may provide a rationale for the future development of blood-based biomarkers for environmental exposure in autoimmune diseases.

## 2. Results

### 2.1. Characteristics of Murine Model Used and Cytokine Profiling

In this study, we used MRL mice exposed to BaP (*n* = 13) or control (*n* = 9) over the course of eight weeks, starting at two months of age. The lungs and skin of the mice were exposed to BaP to model the common absorption of air BaP through the lungs and skin in humans, following established procedures and with a dose (5 mg/kg). We chose this dose because it was at the lower end of the range of doses used in published studies on immune-associated effects of BaP in rodents, i.e., from 3 mg/kg to 90 mg/kg [34,35,36]. Of relevance to this current study, key characteristics of the murine model include a male-specific weight loss at the upon BaP exposure, absence of pathological levels of autoantibody or proteinuria which is consistent with young MRL mice being a pre-autoimmune model, as well as abnormality in cellular and molecular pathways in various organs consistent with BaP-stimulated end organ damage in lupus [26].

In addition to end organs, molecular changes in the blood are of interest to understand in autoimmune studies given that these diseases feature blood abnormalities such as elevation in inflammatory markers [2]. In particular, the abnormal levels of cytokines and their imbalance have been correlated with clinical and blood parameters in various autoimmune diseases including SLE [2]. However, cytokine profiling has been difficult due to the low concentrations (<1 pg/mL) of most proteins in the blood that remain challenging even for highly sensitive immunoassays such as immune-PCR, proximity ligation assay, proximity extension assay and enzyme-linked immunoabsorbent assay [37,38,39].

**Figure 1:**
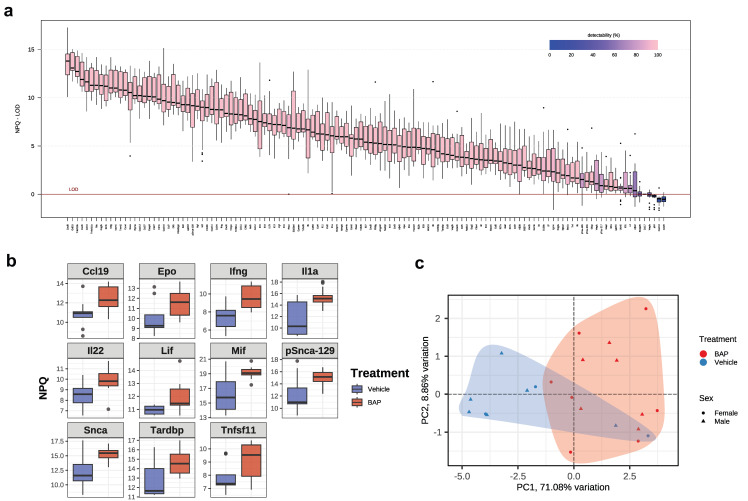
Identification of BaP-responsive serum proteins in MRL mice. (**a**) Boxplot of sample normalized protein quantification (NPQ) distributions by target relative to limit of detection (LOD). (**b**) NPQ levels, which indicate expression levels on the log_2_-scale, of indicated proteins in BaP-treated and control groups. (**c**) PCA plot showing the separation of BaP-treated and control groups.

To this end, we employed the NULISA (NUcleic acid Linked Sandwich Assay) platform, which is known to demonstrate high sensitivity and specificity in detecting biologically important, low abundance biomarkers [28,40]. Using this technique, we found that 94.8% of the total of 123 targets were detectable on this panel, with detectability defined as the % of samples that are above the limit of detection (LOD) (Figure 1a, Supplementary Figure S1a). The targets with low detectability included CCL17 (0%), p24 (0%), CXCL10 (4.5%), CCL20 (9.1%) and VEGFA (45.5%), indicating the low abundance of these cytokines in the MRL serum (Figure 1a). Samples were controlled using the internal control median quality control parameter (Supplementary Figure S1a). The intra-plate coefficient of variation (CV) median was 3.2%, consistent with the reliable quantification of targets across a wide range of abundances.

Comparison between the control and BaP-treated mice identified eleven BaP-stimulated proteins in the serum (*p* < 0.05, Figure 1b). No proteins were found to be significantly downregulated by BaP. These eleven targets distinguished BaP-treated from untreated mice (Figure 1c). Even though the PCA plot does not show complete separation between sexes, we decided to analyze individual cytokine groups separately in male and female mice, considering their known differences in autoimmune disease pathogenesis [41,42,43].

### 2.2. Serum Cytokines Regulated by BaP

**Figure 2:**
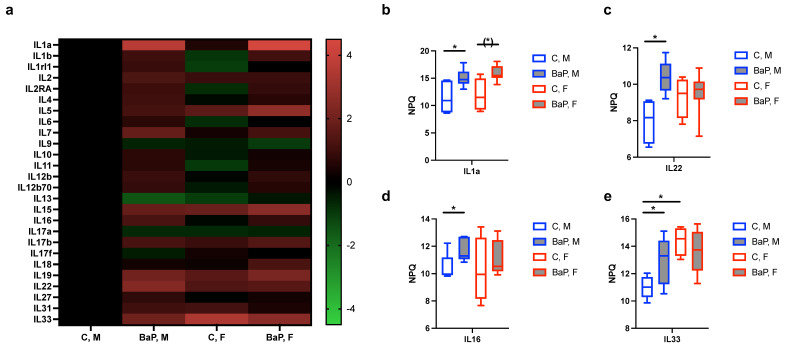
BaP regulation of MRL serum interleukins. (**a**) Heatmap of individual serum interleukin (IL) expression levels in indicated mouse and treatment groups, with a scale bar showing the fold-change in interleukin levels. (**b**–**e**) Normalized protein quantification (NPQ) levels, which indicate expression levels on the log_2_-scale, of IL1A (**b**), IL22 (**c**), IL16 (**d**) and IL33 (**e**) in indicated mouse and treatment groups. C, control. BaP, BaP-treated. M, male. F, female. *, *p* < 0.05, Student’s *t*-test.

Interleukins (ILs) are a group of cytokines critical for mediating immune responses, whose imbalance strongly associates with autoimmune pathogenesis [44,45]. The NULISA panel detected 26 targets in the IL and IL-Receptor Families. The mean of the NPQ (NULISA Protein Quantification) levels, which indicates expression levels on the log_2_-scale, suggests the upregulation of various IL family proteins upon BaP stimulation (Figure 2a). The pro-inflammatory cytokine IL1a showed the most prominent increase upon stimulation, with an NPQ increase of 3.65 in male MRL mice, corresponding to a 12.58-fold change in protein levels (Figure 2b). Other significantly BaP-regulated ILs included IL22, with 5.37-fold increase in males; IL33, with 3.94-fold increase in males; and IL16, with 2.30-fold increase in males (Figure 2b–e), which all have known roles in immune activation [46,47,48,49].

**Figure 3:**
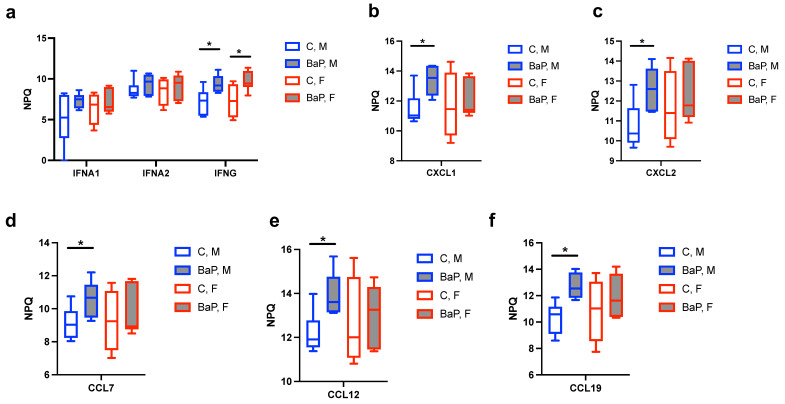
BaP regulation of IFN- and CXCL/CCL-family proteins in MRL serum. Normalized protein quantification (NPQ) levels of IFNA1, IFNA2 and IFNG (**a**), CXCL1 (**b**), CXCL2 (**c**), CCL7 (**d**), CCL12 (**e**) and CCL19 (**f**) in indicated mouse and treatment groups. C, control. BaP, BaP-treated. M, male. F, female. *, *p* < 0.05, Student’s *t*-test.

In addition to ILs, interferon gamma (IFNg) exhibited significant upregulation upon BaP treatment. The increase was observed in both female and male MRL mice, with 6.30-fold and 4.63-fold changes, respectively (Figure 3a). In contrast, whereas the mean level of IFNa2 showed an upward trend with BaP exposure, the change did not reach statistical significance (Figure 3a). This is similar to mercury-induced autoimmunity, in which the key factor was identified as IFNg instead of IFNa [10,11,50].

Of the tumor necrosis factor (TNF) super family proteins, TNFRSF9, which is known to induce the activation of peripheral T cells [51], was found to be significantly upregulated 2.25-fold by BaP in male MRL mice (Supplementary Figure S2).

Of the CXCL and CCL family chemokines, we identified the upregulation of the pro-inflammatory and immune-activating chemoattractants [52,53,54], CXCL1 (3.72-fold), CXCL2 (3.67-fold), CCL7/monocyte chemotactic protein 3 (MCP3, 2.76-fold), CCL12/monocyte chemotactic protein 5 (MCP-5, 3.34-fold), and CCL19/macrophage inflammatory protein-3b (MIP-3b, 5.53-fold). Overall, our data suggests that BaP treatment upregulates protein levels of inflammatory cytokines such as IL1a, IFNg and select TNF and CXCL/CCL family proteins in the MRL blood.

### 2.3. Blood-Based Markers of Neurological Disorders in the BaP-MRL Model

**Figure 4:**
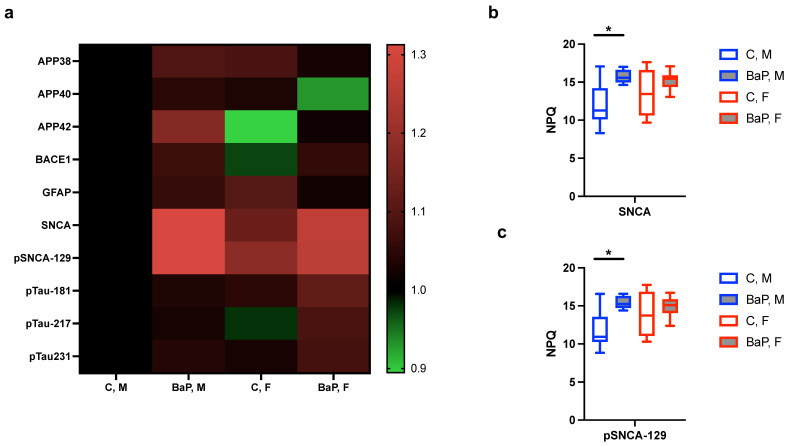
BaP regulation of blood markers for neurological disorder in autoimmunity. (**a**) Heatmap of individual neuronal blood markers in indicated mice and treatment groups, with the scale bar showing the fold-change in protein levels. (**b**,**c**) Normalized protein quantification (NPQ) levels of SNCA (**b**) and its phosphorylated form (**c**) in indicated mouse and treatment groups. C, control. BaP, BaP-treated. M, male. F, female. *, *p* < 0.05, Student’s *t*-test.

Studies have associated systemic lupus erythematosus with cognitive impairment, among additional neuropsychiatric symptoms such as anxiety movement disorders [55,56]. With recent advances in using blood-based markers for the early and sensitive detection of neurodegenerative diseases [57], we measured their abundances in the MRL mice upon exposure to BaP or control. We did not detect significant changes in the Alzheimer disease-specific amyloid or tau pathology markers amyloid-b and p-tau peptides (Figure 4a). However, there was significant upregulation of a-synuclein (SNCA) as well as its pathological, S129-phosphorylated form (Figure 4b,c). These results support the link between neurological abnormalities and lupus and suggest its regulation by exposure to BaP.

### 2.4. Immune Gene Expression in Peripheral Organs

While we detected elevation in the levels of pro-inflammatory proteins in the peripheral blood of MRL mice upon BaP exposure, this does not necessarily mean that peripheral organs also exhibit immune activation in this model. Indeed, autoimmunity is known to be associated with primary immunodeficiency, arguing against a universal overactivation of all immune-associated cell types [58,59].

**Figure 5:**
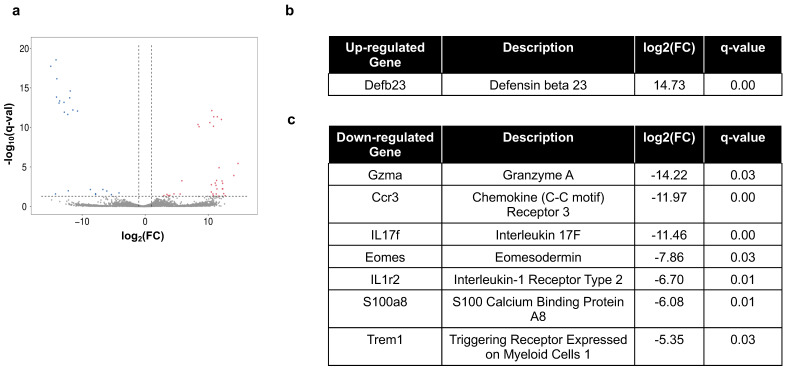
Transcriptomic analysis of BaP-exposed lungs in MRL mice. (**a**) Volcano plot showing fold-change (FC) and q-value on the log-scale for all genes regulated by BaP in the MRL lungs, with red indicating significantly upregulated genes and blue indicating significantly downregulated genes. (**b**,**c**) Immune-associated genes that are significantly up- (**b**) or down- (**c**) regulated by BaP in the MRL lungs.

To understand the BaP regulation of immune-associated genes in the MRL periphery, we performed RNA-Seq experiments of the lungs and skin, two organs directly exposed to BaP in the model (*n* = 5 control and *n* = 6 BaP-treated for each organ). Transcriptomic analysis of the lungs identified 34 upregulated and 22 downregulated genes and with fold-change > 2 and q-value < 0.05 (Figure 5a). Of the upregulated genes, only one gene, defensin beta 23 (Defb23) was a bona fide, immune-associated gene [60] (Figure 5b). In contrast, seven immune-associated genes were downregulated (Figure 5c). Antimicrobial peptides including Defb and S100a8 are known to be expressed by epithelial cells and thus are not expected to be major inflammatory factors in the peripheral blood. Ccl3 and IL17f, however, were included in the blood panel and showed no significant changes upon BaP treatment (Supplementary Figure S3).

**Figure 6:**
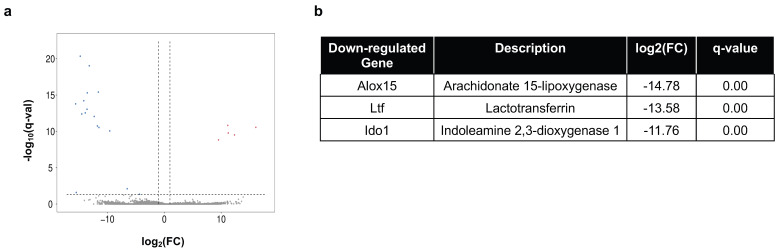
Transcriptomic analysis of BaP-exposed skin in MRL mice. (**a**) Volcano plot showing fold-change (FC) and q-value on the log-scale for all genes regulated by BaP in the MRL skin, with red indicating significantly upregulated genes and blue indicating significantly downregulated genes. (**b**) Immune-associated genes that are significantly downregulated by BaP in the MRL skin.

Transcriptomic study of the skin identified five upregulated and 16 downregulated genes with fold change > 2 and q-value < 0.05 (Figure 6a). Among them, three genes have known immune functions, which were all downregulated (Figure 6b).

Furthermore, we performed qPCR on cytokines known to be expressed in the skin, including IL1a, IL6, IL15, IL18, and IL33 in the MRL model. We found no evidence of immune activation, with the only significantly regulated gene being the inflammatory cytokine IL15, which was decreased by BaP (Supplementary Figure S4). Together, our data suggests tissue-specific immune regulation by BaP in the MRL mice.

## 3. Discussion

We performed a discovery study to identify cytokines that respond to the environmental pollutant BaP in autoimmune-prone animals before disease onset. IL1a, IL22, IL33 and IL16 emerged as leads that were increased by BaP in the peripheral blood. IL1a and IL33 are both IL-1 family proteins, both having broad expression patterns and pleiotropic, inflammatory effects, and contribute to inflammatory and autoimmune disorders [61]. IL22 is made by various T cell subsets, is in the IL10 family but has distinct function in affecting adaptive immunity, inducing the chemotaxis of neutrophils and monocytes as well as facilitating tissue repair [62]. IL22 has been found to participate in the progression of various autoimmune diseases including aggravating lupus nephritis [62,63]. CD16 is expressed by many immune cell types including T cells, B cells, neutrophils and monocytes and acts as a chemoattractant for cells bearing CD4 or CD9 [64]. The role CD16 plays in systemic lupus erythematosus ranges from the gene level, with its polymorphism associating with lupus risk, to the protein level as a biomarker for lupus nephritis [64,65]. Therefore, our data suggests that BaP affects a broad range of peripheral blood cells through specific cytokines in the pre-autoimmune setting.

Intriguingly, while IL1a was found to be upregulated by BaP in the MRL serum, the level of IL1b did not show significant changes. In their secreted form, IL1a and IL1b can bind the same receptors and exert similar biological functions [66,67]. However, the two IL1 molecules show important differences including those in their production, activation, and compartmentalization [66,67]. In the future, it will be of interest to dichotomize the IL1 action by inhibiting IL1b in animal models or in cultured cells, for example.

Historically, type-I IFN is considered the primary pathogenic factor in SLE [68,69]. With clinical trials targeting type-I IFN showing variable results, recent studies support the heterogeneity of SLE disease manifestation and hypothesize that other types of IFN may also play a role [70]. For example, IFN-I, IFN-II and IFN-III fluctuate significantly over time in patients with SLE [71]. Mechanistically, disease processes such as estrogen modulation of endosome-associated toll like receptor 8 (TLR8) in SLE have been shown to be independent of IFNa [72]. Intriguingly, we found that BaP exposure of MRL mice results in the upregulation of serum IFNg but not IFNa1/a2, which is similar to the reported scenario of mercury-induced autoimmunity [9,10]. These results raise the intriguing possibility that exposure to environmental pollution may play a role in affecting the dynamic IFN signature in SLE.

There is emerging evidence supporting neuropsychiatric manifestations of autoimmune diseases. Mechanistically, the C-terminal region of Nedd5 has been identified as a novel autoantigen in SLE with psychiatric manifestations [73]. Neuropsychiatric lupus symptoms have led us to investigate potential blood markers for neurological disorders in MRL mice, and a significant upregulation of SNCA, including its S129-phosphorylated form, was found upon BaP exposure. Neuroinflammation is known to be a main driver of synucleinopathies, with peripheral T cells increasing a-synuclein pathology via modulation of CNS myeloid cell function [74,75]. Our finding is consistent with the hypothesis that smoking and other modes of BaP exposure facilitate T cell activation, thus contributing to the neurological sequelae of lupus.

SLE is strongly sex-biased, affecting women nine times more frequently than men [2,43]. However, the disease is more severe in male patients, with increased risk of disease progression to renal failure and lung cancer, especially in smokers [76,77]. In support of these observations, we have found that exposure to BaP increased many inflammatory cytokines, such as IL22, IL16, CXCL1 and CCL12, in male autoimmune-prone mice. This finding may lead to future studies that further elucidate the role these cytokines play in affecting the disease progression of lupus.

Mechanistically, BaP binds to the Aryl Hydrocarbon Receptor (AhR), leading to its translocation to the nucleus and transcription of downstream genes including cyp1a1 [78]. CYP1A1 mediates the production of the ultimate active metabolite benzo[a]pyrene-7,8-dihydro-diol-9,10-epoxide (BPDE), which can bind DNA and form BPDE-DNA adducts that can subsequently cause DNA damage and/or activate DNA sensors to trigger an immune response [78,79,80,81]. Additionally, BaP has been shown to cause reactive oxidative stress (ROS), causing cellular damage [78,79,80,81]. For example, BaP induces oxidative stress-mediated IL8 production in human keratinocytes via AhR [81]. BaP also contributes to macrophage and astrocyte-mediated neuroinflammation by inducing IL1b and MCP1 production, which is likely to occur through CYB and/or oxidative stress pathways [82]. For future studies, it will be interesting to test whether BaP upregulates cytokines in pre-autoimmune subjects through similar pathways.

Moreover, BaP is known to modify the composition of cholesterol-rich microdomains (lipid rafts) via AhR and BaP-related H_2_O_2_ formation [83]. Lipid rafts are involved in autoimmunity in various ways [84]. The initial events of T-cell activation involve the movement of the T-cell receptor into lipid rafts and the associated aggregation of co-stimulatory molecules [85]. Recent studies describe the pathological remodeling of lipid rafts, leading to the activation of NF-κB and MAPK pathway autoimmune diseases [84,86,87]. Therefore, BaP may cause cytokine dysregulation by altering lipid raft-initiated signaling in autoimmune-susceptible individuals.

One limitation of the study is that we were unable to follow up the mice to the timepoint when they produce autoantibodies or have renal inflammation. Future, longitudinal studies should be designed to connect early cytokine changes with disease onset.

It is worth noting that while BaP may associate with autoimmunity, it is not necessarily a causal association. Indeed, in drug-induced autoimmune hepatitis (DIAIH), an excellent analysis of Teschke et al. showed that 49 drugs may have caused DIAIH with verified diagnoses when potentially confounding, alternative causes were correctly excluded [88]. Autoimmunity in DIAIH develops without BaP having a causal effect. While BaP is widespread and the majority of the human population may be exposed to BaP at some level, we cannot rule out the possibility that autoimmunity can occur in the absence of BaP.

In summary, our study suggests that exposure to BaP leads to cytokine imbalance in a pre-autoimmune model. With the peripheral blood showing a distinct immune profile compared with end organs, our results support the future development of blood-based markers for the early detection of smoking-caused damage in autoimmune-susceptible individuals.

## 4. Material and Methods

### 4.1. Animals

MRL mice were purchased from the Jackson Laboratory and subjected to BaP treatment over the course of 8 weeks starting at 8 weeks of age following established procedures [26,34,35,36]. Mouse body weight was monitored biweekly. At the end of treatment, terminal blood collection was performed for anti-dsDNA antibody ELISA and cytokine profiling. Tissues were snap frozen for RNA analysis. All animal experiments were conducted with IACUC approval.

### 4.2. NULISASeq

Mouse serum was analyzed for multi-protein quantification using the NULISASeq platform following established procedures at Alamar Biosciences [28]. Samples were briefly centrifuged at 10,000× *g* for 10 min, followed by incubation with the capture and detection antibody cocktails. The formed immunocomplex was captured by the dT beads, washed, and further captured by the streptavidin beads. After additional washing, the reporter molecules were eluted and collected for quantification by next-generation-sequencing. For next generation sequencing analysis, the library was cleaned using the AMpure XP reagent (Monroviao, CA, USA) and quantified before being sequenced using the Illumina NextSeq instrument (San Diego, CA, USA). Each sample was identified using a unique sample barcode in the ligator sequence, normalized and analyzed for protein quantification following established pipelines [28].

### 4.3. Transcriptomic Analysis

Upon harvest, mouse tissues were immediately snap frozen using liquid nitrogen and stored at −80 °C before analysis. Total RNA was extracted from snap frozen mouse tissues using the Qiagen RNeasy kit. The integrity of the RNA was quality controlled using Tapestation, passing the RIN criteria. The RNA sequencing library was prepared following the Illumina TruSeq-stranded RNA kit, with ribosomal RNA removed using the Illumina RiboZero rRNA depletion kit. Library was quality controlled with the Agilent Bioanalyzer (Santa Clara, CA, USA) and sequenced on the Illumina NovaSeq 6000 Sequencing System using paired-end sequencing. Sequencing data was quality controlled, read-mapped and expression-quantified, and differentially expressed genes were identified following established pipelines [89,90].

### 4.4. Quantitative Polymerase Chain Reaction (qPCR)

Total RNA was extracted with the Purelink RNA Mini Kit and reverse transcribed using SuperScript IV First-Strand Synthesis System following the manufacturer’s instructions. qPCR reactions were carried out using SYBR Green PCR Master Mix on QuantStudio 7 Flex Real-Time PCR.

qPCR primer sequences are as follows:
IL1a:ACGGCTGAGTTTCAGTGAGACC CACTCTGGTAGGTGTAAGGTGCIL6:TACCACTTCACAAGTCGGAGGC CTGCAAGTGCATCATCGTTGTTCIL15:GTAGGTCTCCCTAAAACACAGAGGC TCCAGGAGAAAGCAGTTCATTGCIL18:GACAGCCTGTGTTCGAGGATATG TGTTCTTACAGGAGAGGGTAGACIL33:CTACGCATGAGACTCCGTTCTG AGAATCCCGTGGATAGGCAGAG

### 4.5. Statistical Analysis

Unless otherwise indicated, Student’s *t*-test (two-sample, unequal variances) was used to compare experimental (knockdown or inhibition) versus control groups [91,92,93]. The choice of statistical test was based on textbook knowledge and up-to-date literature [91,92,93,94,95]. Values in bar graphs were shown as mean ± s.e.m, with *p* values less than 0.05 indicated with asterisks.

## Data Availability

Data supporting this study is available upon request.

## Supplementary Materials

The following supporting information can be downloaded at https://www.mdpi.com/article/10.3390/ijms262010242/s1.
