# Supplementary material for: Discovery of Blood-Based Proteins That Mark Benzo[a]pyrene Modulation of Autoimmunity
Source: Int J Mol Sci. 2025 Oct 21;26(20):10242. doi: 10.3390/ijms262010242 (PMC12565278; doi:10.3390/ijms262010242)
Supplement: Supplementary file 1 [file ijms-26-10242-s001.zip › ijms-3920064-supplementary.pdf]

## Supplementary Figure 1

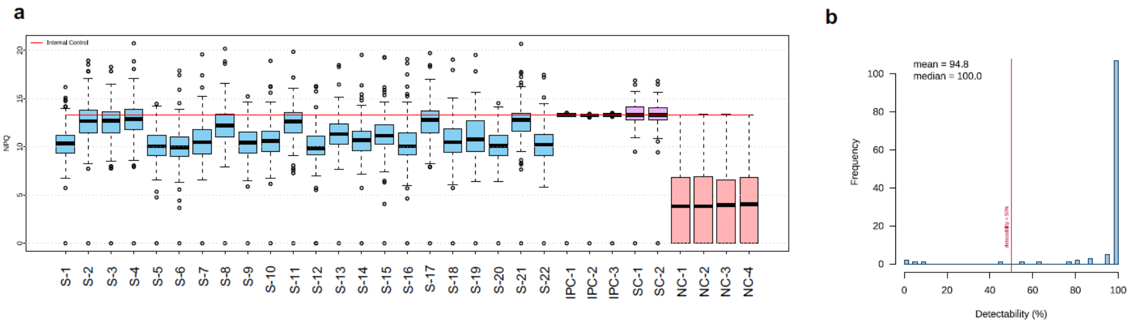


**Figure S1. Quality control of the NULISA panel.** (a) Normalized protein quantification (NPQ) levels of targets are shown for the testing samples (S), interplate control samples (IPC), internal sample controls (SC), and negative controls (NC). (b) Detectability plot of targets, showing a mean detectability of 94.8% and median detectability of 100% in all samples.

## Supplementary Figure 2

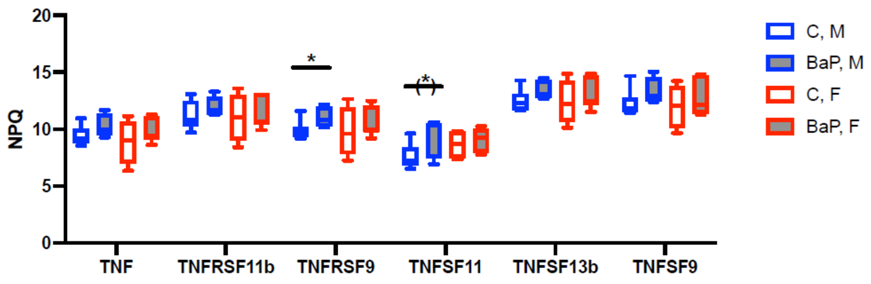


**Supplementary Figure S2. BaP regulation of the TNF- and receptor super family proteins in MRL serum.** Normalized protein quantification (NPQ) levels of TNF, TNFRSF11b, TNFRSF9, TNFSF11, TNFSF13b and TNFSF9 in indicated mouse and treatment groups. C, control. BaP, BaP-treated. M, male. F, female. *, $P < 0.05$, Student's t-test.

## Supplementary Figure 3

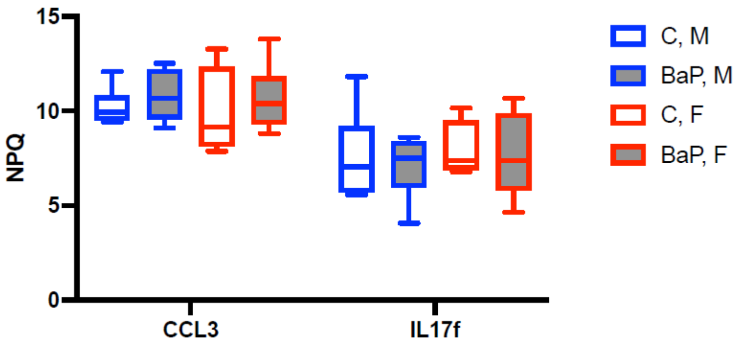


**Supplementary Figure S3. Blood CCL3 and IL17f levels upon BaP treatment.** Normalized protein quantification (NPQ) levels of CCL and IL17f in indicated mouse and treatment groups. C, control. BaP, BaP-treated. M, male. F, female.

## Supplementary Figure 4

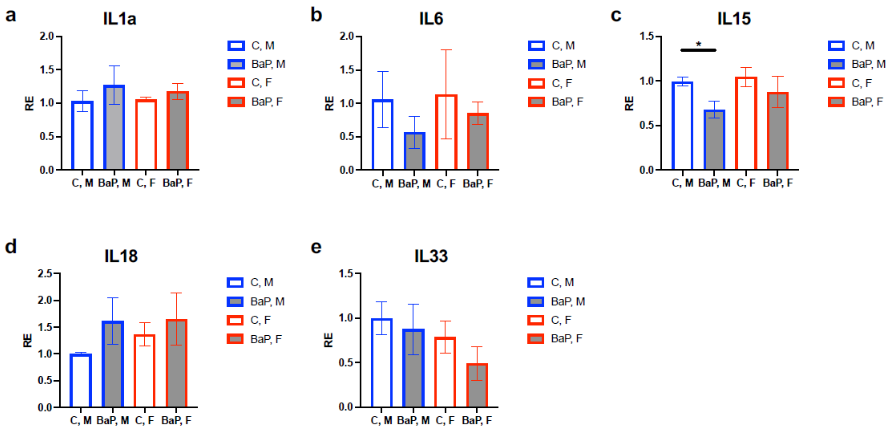


**Supplementary Figure S4. Skin interleukin expression levels upon BaP treatment.** Relative expression (RE) levels of IL1a (a), IL6 (b), IL15 (c), IL18 (d) and IL33 (e) in the skin in indicated mouse and treatment groups. C, control. BaP, BaP-treated. M, male. F, female. *, $P < 0.05$, Student's t-test.
